# Structural determination of Enzyme-Graphene Nanocomposite Sensor Material

**DOI:** 10.1038/s41598-019-51882-7

**Published:** 2019-10-29

**Authors:** Durgesh K. Rai, Manickam Gurusaran, Volker Urban, Kiana Aran, Lulu Ma, Pingzuo Li, Shuo Qian, Tharangattu N. Narayanan, Pulickel M. Ajayan, Dorian Liepmann, Kanagaraj Sekar, María-Efigenia Álvarez-Cao, Juan-José Escuder-Rodríguez, María-Esperanza Cerdán, María-Isabel González-Siso, Sowmya Viswanathan, Ramasamy Paulmurugan, Venkatesan Renugopalakrishnan

**Affiliations:** 1 Cornell High Energy Synchrotron Source, Cornell University, Ithaca, New York 14853 USA; 2 Institute for Cell and Molecular Biosciences, Newcastle University, Newcastle upon Tyne-NE1 7RU, UK; 3 Neutron Scattering Division, Oak Ridge National Laboratory, Oak Ridge, Tennessee 37831 USA; 4 Department of Bioengineering, University of California, Berkeley, Berkeley, California 94709 USA; 5 Department of Mechanical Engineering and Materials Science, Rice University, Houston, Texas 77005 USA; 6 Center for Life Sciences, Boston Children’s Hospital, Harvard Medical School, Boston, Massachusetts 02115 USA; 7 Tata Institute of Fundamental Research – Center for Interdisciplinary Sciences, Hyderabad, 500107 India; 8 Department of Computational and Data Sciences, Indian Institute of Science, Bangalore, 560012 India; 9 Universidade da Coruña, Grupo EXPRELA, F. Ciencias & Centro de Investigacións Científicas Avanzadas (CICA) & Instituto de Investigación Biomédica A Coruña (INIBIC), A Coruña, 15011 Spain; 10 Newton Wellesley Hospital/Partners Healthcare System, Newton, Massachusetts 02462 USA; 11 Cellular Pathway Imaging Laboratory (CPIL), Dept. of Radiology, Stanford University School of Medicine, 3155 Porter Drive, Suite 2236, Palo Alto, California 94304 USA; 12 Department of Chemistry and Chemical Biology, Northeastern University, Boston, Massachusetts 02115 USA

**Keywords:** Biomedical materials, SAXS

## Abstract

State-of-the-art ultra-sensitive blood glucose-monitoring biosensors, based on glucose oxidase (GOx) covalently linked to a single layer graphene (SLG), will be a valuable next generation diagnostic tool for personal glycemic level management. We report here our observations of sensor matrix structure obtained using a multi-physics approach towards analysis of small-angle neutron scattering (SANS) on graphene-based biosensor functionalized with GOx under different pH conditions for various hierarchical GOx assemblies within SLG. We developed a methodology to separately extract the average shape of GOx molecules within the hierarchical assemblies. The modeling is able to resolve differences in the average GOx dimer structure and shows that treatment under different pH conditions lead to differences within the GOx at the dimer contact region with SLG. The coupling of different analysis methods and modeling approaches we developed in this study provides a universal approach to obtain detailed structural quantifications, for establishing robust structure-property relationships. This is an essential step to obtain an insight into the structure and function of the GOx-SLG interface for optimizing sensor performance.

## Introduction

Biohybrid composites that integrate biological functionality with the nanoscale organization of inorganic compounds hold great promise for designing materials that take inspiration from nature to combine biological or biomimetic functional components with man-made synthetics^1,2^. These complex materials often strive to emulate hierarchical architectures that are typical of natural biological systems^3,4^. It is challenging to precisely manufacture such detailed structures, and one major limitation lies in the availability of imaging methods that are needed for determining the detailed nanoscale organization of a manufactured biohybrid materials and then using this information for optimal manufacturing of materials with required parameters. Here, we present our investigation into a new generation of graphene-based glucose biosensors where accuracy of structural organization is of prime concern for the sensor functionality. We relate the performance of this sensor to detail the nano-scale structure. We also describe a new approach for extracting the structural details from small-angle neutron scattering data.

The graphene-based sensors are expected to aid direct and effective electron transfers by eliminating the use of redox mediators and oxidation overpotential^5–8^. Graphene, a two dimensional single-atom-thick crystalline nanomaterial, has engaged a wide range of markets due to its superlative qualities^9^. Unlike semiconductors that are reaching fundamental limitations for device fabrication, graphene based electronic devices hold an immense potential for future electronics^6,8,10^. Proteins confer exceptional stereospecificity to graphene when covalently attached in the design of biosensors. Glucose oxidase (GOx), a redox enzyme that increases the rate of glucose oxidation, has been extensively employed in biosensors to determine the concentration of glucose in biofluids by estimating the number of electrons released during the redox reaction^11–15^. GOx in conjunction with graphene interface acts as an efficient conveyor belt, receiving the electrons from flavin adenine dinucleotide (FAD) cofactors in GOx and ballistically transporting them as output, to give a direct quantitative measure of glucose in biofluids^8^. The graphene-protein interface is the gateway through which electrons shuttle from the FAD centers in GOx^4^.

Structural details of immobilized GOx on pristine graphene are hard to derive, and at present, the repertoire of structural methods are available to investigate interfaces which are largely limited to in silico molecular dynamics^4^, small-angle scattering, and 3D NMR imaging. Small-angle neutron scattering (SANS) probes structural features in the region from 1 nanometer to hundreds of nanometers. The prime objective of the present SANS study is to establish a detailed understanding of the GOx matrix structure deposited on graphene and relating the structural modifications to different sample conditions, and in future relating this information with the functionality of sensors in different applications.

Recent enhancements in capabilities of small-angle scattering techniques for biological samples have occurred in parallel with new methods for analyzing and modeling of data^16,17^. SANS provides unique capabilities to determine the organization and hierarchical structures of different characteristic length scales in a single measurement^18^. In the present study we have applied a novel scaling approach to Unified Fit^19^ based analytical method to simultaneously extract the structure of individual GOx macromolecules in the biosensor and the architecture of assemblies that it forms within the sensor material. We use the Unified Fit in combination with *ab initio* methods provided by tools in the openly available ATSAS software suite and rigid body docking to obtain biologically relevant structural models for different structural levels^20,21^. The scaling approach overcomes the limitations of *ab initio* models in order to evaluate average models of the hierarchical assemblies. The results of this study demonstrate the ability of SANS to differentiate between changes in hierarchical structures in sensor matrices, which correlates well with the individual manufacturing processes.

**Figure 1:**
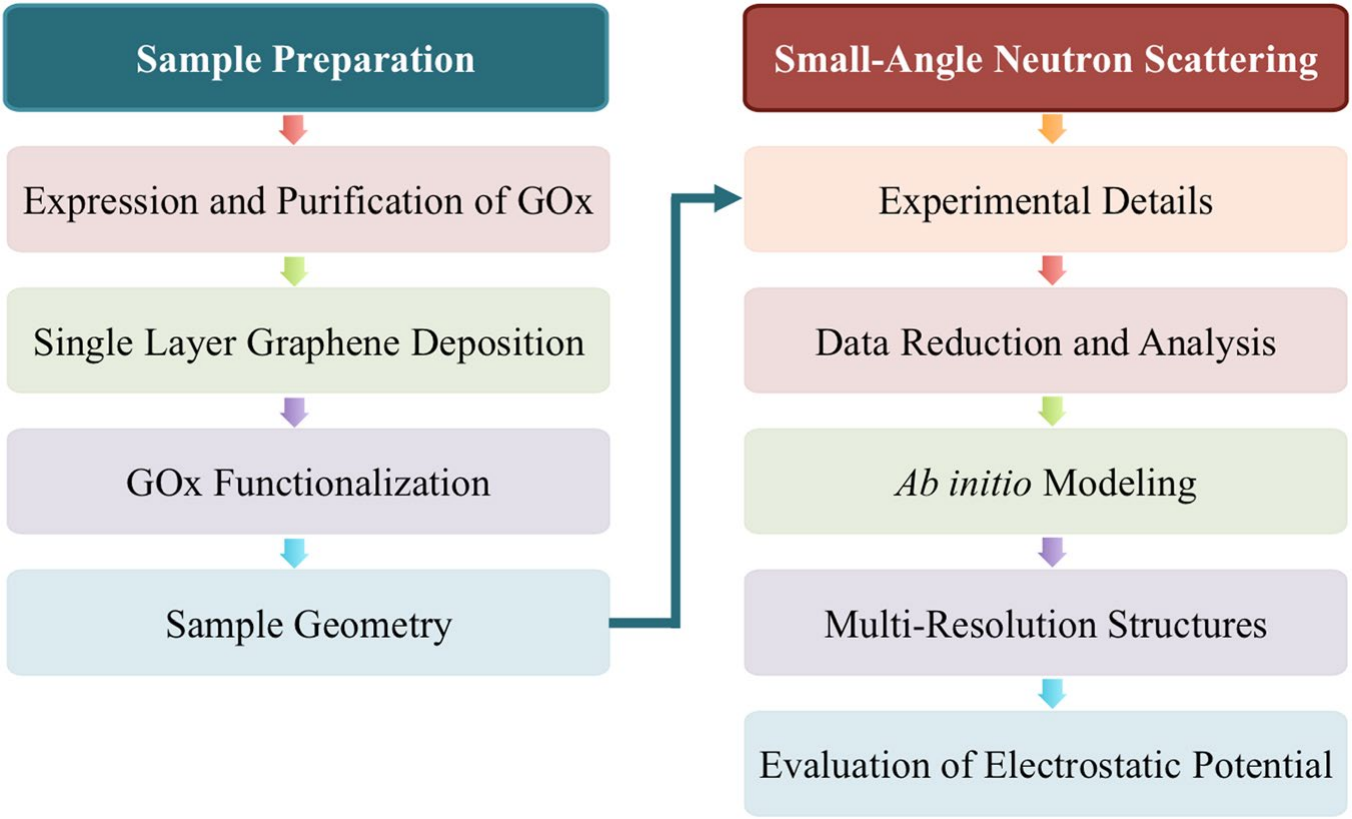
Schematic work-flow of experiments performed for the study. The samples composed of functionalized GOx matrix that is investigated using a multi-physics approach for SANS data analysis as discussed.

Figure 1 shows a brief layout of the approach for sample preparation and data analysis. The details of the sample preparation are discussed in the beginning of Materials and Methods section. It is followed by the details of small angle neutron scattering methods and data analysis. The next section discusses the information extracted from the SANS results and *ab-initio* models, that are compared with the electrostatic potential on the GOx crystal structure evaluated using continuum electrostatics calculations.

## Results and Discussions

**Figure 2:**
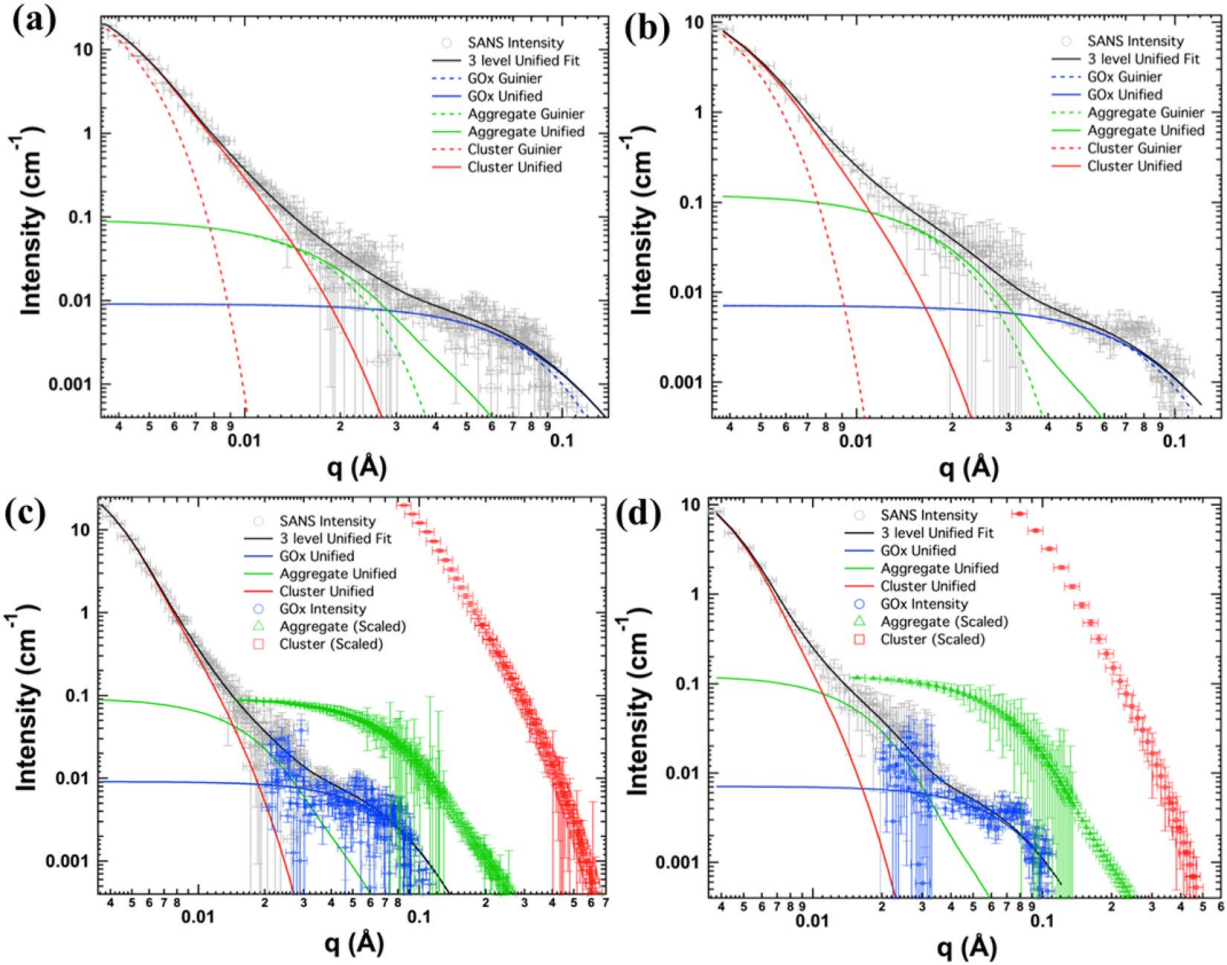
SANS data in grey circles from (**a**) sample 1 and (**b**) sample 2 with the three level Unified Fits (solid black line) with the individual level fits shown in solid and Guinier fits in dashed, blue, green and red solid lines for GOx, aggregate and cluster levels respectively. The same data from sample (**c**) 1 and (**d**) 2 with the three level Unified Fits, showing the contribution from each level in solid blue (GOx level), green (aggregate level) and red (cluster level) lines. The corresponding scaled contribution using Eqs (6–10) are shown in blue circles, green triangles and red squares.

We have investigated the structural details of GOx-graphene sensor material on a length scale spanning 1–200 nm by small-angle neutron scattering (SANS). Figure 2(a,b) shows SANS data for two samples prepared by incubating graphene with recombinant GOx in buffer solution at pH 7 and pH 9, respectively. The scattering intensity shows several distinct bends which were analyzed with the help of a 3-level Unified Fit using Eq. (1)^19^. This analysis approach yields a set of parameters that describe the average topological arrangement at each level of the hierarchical structure. The parameters extracted from these fits are given in Table 1. We discuss the significance of these structural parameters in the following section, beginning at the smallest level of structure.

**Table 1:** Fitting parameters from 3-level Unified Fit, i.e. intensity scale factors G_i_, B_i_, gyration radii for the 3 structural levels R_g,i_, and power law scaling exponent for the intermediate structural level, d_f_. From the ratios of intensity scales G_i_ are derived the number of subunits within one unit of the next higher structural level; i.e. z_a_ GOx dimers form on average one aggregate unit, and z_c_ aggregates compose one structural cluster.

Sample	G_G_ (cm^−1^)	G_a_ (cm^−1^)	G_c_^*^ (cm^−1^)	B_a_ (cm^−1^Å^−d^_f_)	B_c_^*^ (cm^−1^Å^−4^)	R_g,G_ (Å)	R_g,a_ (Å)	R_g,c_ (Å)	z_a_	z_c_	d_f_
1	91 ± 5 x10^−4^	9 ± 1 x10^−2^	86 ± 4	1.7 ± 0.1 x10^−6^	43.3 ± 0.9 x10^−10^	25.5 ± 0.6	110 ± 10	590 ± 20	10 ± 2	920 ± 90	2.34 ± 0.07
2	71 ± 4 x10^−3^	12 ± 1 x10^−2^	33 ± 3	2.6 ± 0.2 x10^−7^	23.6 ± 0.6 x10^−10^	26.2 ± 0.3	108 ± 6	550 ± 30	17 ± 2	250 ± 30	2.86 ± 0.05

A size parameter in the form of apparent radius of gyration, *R*_*g*_ of 25 to 26 Å is observed in both samples at the largest measured scattering angles (*q* > 0.04, see materials and methods for the relation between scattering angle, scattering vector *q*, and observed length scale). This size matches with the length scale of individual GOx enzymes and interpretation of this feature as stemming from GOx enzymes is therefore very plausible. The overall envelope shape of individual enzyme molecules in solution can successfully be obtained from small-angle scattering data^21^. For a dense packing of enzymes as encountered in the GOx sensor material, such a detailed analysis is obstructed by the additional scattering features that arise from the higher-levels of structure. In order to overcome this limitation, we have subtracted the higher-level scattering features, which were determined by their corresponding 3-level fit parameters. The residual scattering was then used for generating shape models of GOx enzyme by applying tools from the ATSAS suite as described in detail in the methods section^22,23^.

**Figure 3:**
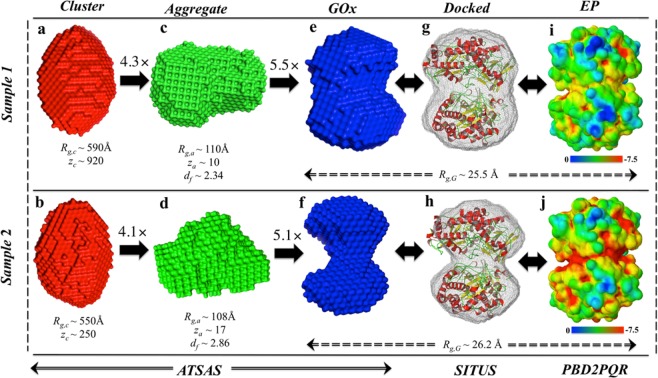
Schematic diagram of topological details of sample 1 and 2 with varied fractal and aggregate features using models obtained through ATSAS (**a**–**f**), SITUS (**g**,**h**) and PDB2PQR (**i**,**j**), as depicted at the bottom. Low-resolution models (surface) using Eqs (6–10) as basic functions in ATSAS suit for (**a**) sample 1 and (**b**) sample 2 clusters; (**c**) sample 1 and (**d**) sample 2 aggregates and (**e**) sample 1 and (**f**) sample 2 GOx dimers. The ratios of upper to lower level ${R}_{g,i}\text{'}s$ were used to zoom in into the structure, shown on top of arrows with $({R}_{g,i}/{R}_{g,i-1})\times$. An exhaustive 6D conformational fitting was preformed on the high-resolution crystal structure into the modeled low-resolution averaged molecular envelopes to derive the docked complexes of sample 1 (**g**) and sample 2 (**h**). Comparison of surface electrostatic potentials under pretreatment conditions of pH 7 for sample 1 (**i**) and pH 9 for sample 2 (**j**). The blue-green-red linear color scale below (**i**) and (**j**) represents EPs in units of k_B_T/e (T = 298.15 K).

The final DAMAVER density maps for GOx enzymes in the sensor matrix of sample 1 and 2 are shown as blue surface in the central column of Fig. 3(e,f). GOx models for both the samples show a narrowed waist region in the center21,22. This feature is more prominent in sample 2. A comparison of these shape models with the known crystal structure of GOx is shown in Fig. 3(g,h), which depicts a quantitative rigid-body docking of the high-resolution structure into the low-resolution DAMAVER density map of sample 1 and 2 respectively. The resulting docked complex was inspected and visualized using *PyMOL*^24^. Apparently, the GOx dimer high-resolution structure, which was obtained through crystallizing GOx from a dilute solution, describes the conformation of GOx in the graphene sensor material rather well, especially for the sensor material prepared at neutral pH 7. Intriguingly, the sensor material that was prepared at basic pH 9 shows a somewhat increased separation between the subunits—note that some residues on the left side of Fig. 3(h) do not fit well into the more separated envelope structure.

Since the differences in sample preparation are in the pH environment, the pronounced neck region for sample 2 can be further examined using electrostatic potential (EP) evaluation employing continuum electrostatics calculations, shown in Fig. 3(i,j). The output has been generated using Jmol by *PBD2PQR* on a linear red-green-blue colour scale representing EPs of −7.5−0*K*_*B*_*T*/*e*. The EP over the structure differs markedly in the neck region of the dimer structure. The marked differences in the electrostatic potential distribution in the neck region may alter the electrostatic interactions at the dimer interface, and hence, induce greater separation of the dimer subunits at the elevated pH level^25^. Importantly, changes of the electrostatic potential may lead to altered glucose recognition and loss of its catalytic abilities. We note that this spatial separation is permanently retained in the final sensor material, since the SANS data was acquired after washing the material with neutral buffered saline solutions after functionalization at higher pH. The graphene functionalized GOx sensor material is organized into nano- to mesoscale clusters with an average diameter of just above 1000 Å. This is evident from the observed gyration radii at low scattering angles (*q* < 0.01 Å). The Unified Fit here yields overall size parameters *R*_g_ of 590 Å and 550 Å for sample 1 and 2 respectively. The steep drop-off in intensity, which was fit by a *q*^−4^ power-law supports the notion of 3-dimensionally extended dense clusters that are bound by a well-defined surface. The inferred dimension and nature of the clusters compare well with the reported ESEM micrographs of surface morphology of immobilized GOx on chitosan supported mesoporous carbon^26^ and cross-linked enzyme aggregates of horseradish peroxidase and GOx reported previously^27^. The limitations of the surface morphology information from the ESEM micrographs are testimony to the utility of the present work where (submicron) cluster morphology is deciphered using SANS.

In this same context, it is appealing to extend the envelope shape modeling approach to the larger structural features of the GOx sensor material. Such an approach is generally not possible with modeling software that is optimized for the smaller scales of enzyme structures. We introduced here a new approach to circumvents this limitation. We used structural models obtained from SANS using the ATSAS dummy atoms model (DAM) where the shape was calculated from the individual level scattering contributions obtained from the 3-level Unified Fit using Eqs (6–8). As shown in Fig. 2(c,d), we rescaled the two larger-scale structural levels along the *q*-axis to match the overall size of the GOx dimer using Eq. (10). This step of demagnification is necessary to allow fitting the larger structural levels into the limits of the volume of the search box of the dummy atom-modeling program. Of course, this approach implies a coarsening of the model resolution with increasing levels of structural hierarchy, such that the size of details as represented by the rescaled dummy ‘atoms’ of each structural level is proportional to the overall size of that structural level.

The dummy ‘atom’ models obtained for the clusters look similar for both samples as shown in envelope structures in Fig. 3(a,b). We emphasize that the cluster structure models are derived from scattering intensities as parameterized by the respective Unified Fit curves and taking into account experimental error bars using Eqs 7 and 10. These models consequently do not contain more information than what is represented in the Unified Fit parameters. Moreover, the DAMMIN algorithm was developed for compact globular proteins and utilizes a built-in bias that favors connected, compact structures. Generally, the conversion of a scattering intensity curve in to a 3-dimensional real space model does not have a unique solution. Thus, the models generated by our new scaling approach need to be interpreted cautiously. Even so, the generated envelope structures are valuable as an intuitive illustration of plausible real-space structural features which are consistent with the SANS data.

Independent of any models, the SANS data allows in estimating additional quantities of interest: The ratio of absolute scattering intensity of the cluster to that of the individual GOx enzyme gives an estimate of the number of GOx dimers in the cluster. This number is about 9500 in sample 1 and 4600 in sample 2. Moreover, from the comparison of the size parameters of the cluster and GOx dimer we can estimate that the cluster volume is approximately 12400 times larger than the GOx volume in sample 1 and 9300 times larger than in sample 2. From this we can estimate a packing density of GOx enzymes of 0.76 in sample 1 and 0.50 in sample 2. These are rough estimates as uncertainties accumulate in the combination of fit parameters and anisometric shape of particles was not taken explicitly into account when estimating volume scaling from R_g_ scaling. Nonetheless, these results suggest that GOx is packed quite densely in the sensor material prepared at neutral pH while preparation at higher pH leads to a somewhat less dense sensor material at the nanoscale. A lower packing density in the material prepared at pH 9 is consistent with repulsive surface charges on the GOx discussed above. Lower packing density of the sensor material provides a second plausible explanation for the lower performance observed in sensors prepared at higher pH.

Additional details of the spatial arrangement of GOx molecules inside clusters of graphene sensor material can be extracted from the central region of the scattering curves $(0.01 < q < 0.04)$. The 3-level fit represents here mass fractals of similar overall size ${R}_{g}$ of 108 to 110 Å for both samples but with differing power law exponents ${d}_{f}$ of 2.34 and 2.86 respectively. The presence of this intermediate length scale of scattering signifies additional spatial correlations in the material that would not be observed by a simple, homogeneous, random arrangement of GOx within the large clusters of the material. Rather, it signifies specific structural arrangement at an intermediate scale. This feature is more pronounced in sample 2 prepared at pH 9. The 3-level Unified Fit implies that this intermediate scattering level represents aggregates composed of the smaller scale level GOx dimers. Following this interpretation, we find the number of GOx enzyme molecules in these mass-fractal aggregates, *z*_*a*_, evaluated from the absolute scattering intensity to be 10 and 17 for sample 1 and sample 2 respectively. On the other hand, if we assume that volume scales as ${R}_{g}^{3}$, then we find that the volume spanned by the aggregates is 70 to 80 times larger than that of GOx, which leads to a low space filling of GOx molecules of 0.12 and 0.24 for sample 1 and 2 respectively.

On the other hand, the comparison of size parameters of aggregates and the larger clusters yields large numbers of several hundred aggregates within a cluster (*z*_*c*_, Table 1), which would require packing fractions greater than 1 (6 and 2 for sample 1 and 2 respectively). A packing fraction of 1 represents complete space filling and numbers greater than 1 are not physically feasible. One possible explanation for this observation is that aggregates are not isometric but rather spatially extended in one or two dimensions, such as forming rods or platelets. Scaling as ${R}_{g}^{3}$ would then grossly misrepresent the aggregate volume. In this scenario, ${R}_{g}$ would initially grow fast as GOx packs for example into aggregate platelets, and then slow, as platelets stack to form clusters, in agreement with the observed SANS data. Platelets would exhibit a scaling exponent ${d}_{f}=2$ in the *q*-range that corresponds to length scales larger than their thickness but smaller than their radius^28^. The unified fit yields power law exponents ${d}_{f}$ of 2.34 and 2.86 which are not quite consistent with platelets but which would allow anisometric fractal objects that grow slower in volume than ${R}_{g}^{3}$. The lower exponent for sample 1 suggests that such aggregates may be more flattened for samples prepared at neutral pH rather than basic pH.

Dummy atom models of aggregates representing the intermediate structural level are shown in Fig. 3(c,d), which is composed of ${z}_{a}$ GOx dimers. As pointed out earlier, these envelope models should not be overinterpreted. Nonetheless, the differences in the morphology of the two samples, as expected from their respective fractal exponents ${d}_{f}$, clearly stand out in these models. Sample 1 exhibits a thick two-dimensional fractal structure, while sample 2 points towards a more branched, 3-dimensionally packed structure that encompasses a considerable concentration of cavities. Importantly, the presence of pores in sample 2 is consistent with the observation presented earlier that clusters in sample 2 are less densely packed with GOx than in sample 1.

Low aggregate mass density structures typically show lower conductivities^29–32^ due to the presence of lower connectivity^18,33,34^. A general dependence of resistivity on fractal topology can be evaluated using simple Cates scaling theory^35^, which would appear to be appropriate in the context of the present investigation. For example, a random walk upon a lattice of native mass fractal dimensions of ${d}_{f}$, results in a path of fractal dimension ${d}_{f}/2$. Then the resulting resistances can be expected to scale as $\Omega (R) \sim 1/{R}^{{d}_{f}/2}$. This confirms that the resistivity contribution from aggregate arrangement would typically be higher for lower fractal dimension structures.

In conclusion, we have shown here the use of SANS to probe structural features of GOx proteins in solid form of sensor matrices. The methodology to generate representative spatial models for the hierarchical structures of enzymes on glucose sensor surface by combining Unified Fit analysis with the widely used ATSAS suite has been demonstrated. The overall performance of the sensor material is expected to result from a combination of the average enzymatic functionality of matrix-embedded GOx dimers and the conductance of graphene-functionalized GOx topologies at different length scales. This work demonstrates the power of SANS-based analysis for obtaining structural information that can help understand these competing factors in bio-composite sensor materials. The modeling methodology introduced here can be generically used in device configurations or other bio-macromolecule based functional materials. The methodology brings to light some unique opportunities for the application of SANS to predict or infer the performance of bio-hybrid sensor matrices in solid form on complex substrates.

## Materials and Methods

### Recombinant GOx expression and purification

The gene encoding a glucose oxidase from *Aspergillus niger* BT18 (Target species: ASPNG; Uniprot ID P13006) was cloned into a replicative plasmid vector and expressed in *Kluyveromyces marxianus var. marxianus* CBS 6556 strain as described previously^36^. A partially purified enzyme (Medium grade GOx) obtained from cell-free supernatant prepared by culture centrifugation (13,000 rpm, 30 mins, 4 °C) was concentrated and exchanged by Prep/Scale Spiral Wound Ultrafiltration with a pore size of 10 kDa (Millipore) in 20 mM Tris buffer (pH 7.4) with 100 mM NaCl. The pure enzyme (High grade GOx) was subjected to a second purification step with Q-Sepharose F.F. column (strong anion exchanger) equilibrated with the same buffer, and a linear gradient of 0–0.5 mM NaCl in 20 mM Tris buffer (pH 7.4) was used to elute the column at a flow rate of 0.5 ml.min^−1^. Enzyme activity recovery and purification factor were 89% and 1.5 for the Medium grade GOx, and 17.3% and 17 for the High grade GOx. Both Medium grade and High grade GOx were lyophilized to achieve higher stability at room temperature. All steps of purification were assayed for their enzyme activity and protein content. GOx’s activity was determined by the o-dianisidine reduction method using a commercial glucose oxidase from *Aspergillus Niger* (G6125, Sigma, USA) as standard at 37 °C and pH 4.5^37^. The homogeneity of the partially and highly purified protein was confirmed by SDS–PAGE analysis (Fig. S1) and the protein concentration was determined by Bradford method using BSA as standard^38,39^.

### Single layer graphene deposition

Single layer graphene film was synthesized on a 25 μm thick clean copper (Cu) foil by the chemical vapor deposition technique using hexane as the carbon source^40^. The Cu foil was loaded in a quartz tube and the tube was pumped down to 10^−2^ Torr using a vacuum pump, before flowing in Ar/H_2_ mixture gas at a pressure of ∼8–9 Torr with a flow rate of ∼400 standard cubic centimeters per minute (sccm). The Cu foil was then heated to 950 °C in Ar/H_2_ atmosphere. When the temperature becomes 950 °C, Ar/H_2_ flow was stopped and hexane vapor is passed through the quartz tube maintaining the tube pressure of 500 mTorr for 4 min. The flow rate of hexane was maintained at ~ 4 mL/h (~2–3 sccm). The furnace was then suddenly cooled to room temperature. The graphene growth has taken place over Cu foil and this film was further processed for PMMA based transfer process.

### Functionalization of GOx

A graphene device (CVD Graphene or Graphene Foam) was incubated with a 5 mM linker molecule (1-pyrenebutanoic acid succinimidyl ester, Santa Cruz Biotechnology, SC-213409) in dimethylformamide (DMF) for 2 hours at room temperature and washed with pure DMF and deionized (DI) water for three times. The linker-modified graphene was then incubated with 10 mg/mL of recombinant GOx in Na_2_CO_3_-NaHCO_3_ buffer solution (pH 7.0 for Sample 1 and pH 9.0 for Sample 2) overnight at 4 °C followed by rinsing with DI water and phosphate buffered saline solution (PBS, pH 7.2). To deactivate and block the excess reactive groups remaining on the surface, the device was further incubated with 0.1 M ethanolamine solution (pH 7.0 for Sample 1 and pH 9.0 for Sample 2) for 30 min and then rinsed with DI water. Raman spectrum of GOx immobilized on single layer graphene (SLG) collected at ~ pH 7 (blue), after subtraction of SLG, is shown in Fig. S2, which shows well-resolved amide I, III regions, confirming that GOx was successfully immobilized on Graphene surface. The enhancement of the G band assigned for the sp^2^ carbon is an indication of reconstruction of the carbon network after immobilization of GOx on the graphene surface^26^.

### Sample geometry

**Figure 4:**
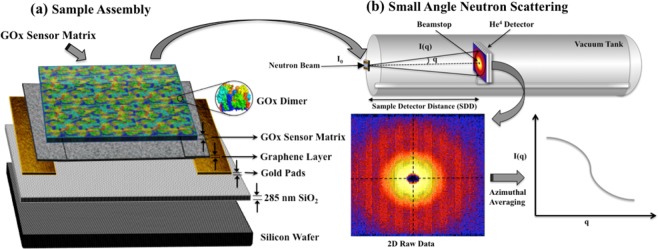
(**a**) Sample assembly of active matrix area under SANS investigation, and (**b**) Sample assembly with an active matrix area sandwiched between quartz slides and placed in Aluminum holders for SANS data acquisition. SANS experiment was set up with sample mounted in transmission geometry at Bio-SANS.

The device has a stack of single layer graphene sandwiched between two gold pads deposited on top of Silicon Dioxide layer on a silicon wafer. A few microns thick layer constituting of an estimated 600–1,000 layers of functionalized GOx deposited over the single layer graphene layer constitutes the activated matrix investigated using SANS. A schematic of the active matrix under investigation is shown in Fig. 4(a).

### Small-angle neutron scattering

#### SANS experiment

SANS experiments were performed at the Bio-SANS instrument at the High-Flux Isotope Reactor in Oak Ridge National Laboratory^41^. The instrument schematic is shown in Fig. 4(b). The sample, GOx sensor matrix was held on a silicon wafer by sandwiching between two quartz slides. The GOx sensor matrix was positioned to have the neutron beam transmit as used for transmission mode measurements. A 2D position sensitive neutron detector housed in a vacuum tank acquired the scattered neutrons carrying structural information of the protein. Three different instrument configurations of sample-to-detector distances (SDD) of 1.13 m, 6.83 m and 15.33 m were employed to acquire SANS data in a wide range of positions (0.003 Å^−1^ < *q* < 0.5 Å^−1^), at a wavelength of 6 Å with a wavelength spread, Δλ/λ of 0.15. The data acquisition time was 5 hours for each sample at each SDD configuration.

The SANS $I-q$ profile was obtained by performing an azimuthal averaging of the 2D detector images on a 256 × 192 pixel matrix, normalized to incident beam monitor counts, corrected for detector dark current, pixel sensitivity, sample transmission and silicon wafer with SiO_2_ and gold depositions as background.

### Small-angle neutron scattering data reduction and analysis

In isotropic small-angle scattering as in present case, an azimuthally averaged intensity in units of cm^−1^ is plotted against a reduced angle, called the scattering wave vector *q*. $q=\frac{4\pi }{\lambda }Sin\theta$, where, *λ* is the wavelength and 2*θ* is the scattering angle. SANS data can be used to quantify topological features in complex materials^20,42^. SANS from a hierarchical system displays multiple structural levels which can be evaluated using scattering laws for mass/size and surface/density properties^2,18,43,44^. In each structural level, a Guinier’s law, $I(q) \sim Gexp(-{q}^{2}{R}_{g}^{2}/3)$_,_ and a power-law, $I(q) \sim B{q}^{-{d}_{f}}$, are observed at the lower and higher *q-*regions respectively, where *G*, *R*_*g*_, *B* and *d*_*f*_ are the Guinier’s law prefactor, radius of gyration, power-law prefactor and fractal dimension respectively^18,43–45^. A Guinier-law is an exponentially decaying function, which decays near *qRg* = 1 region and the actual shape of the decay, also known as the Guinier knee, depends on the shape and size of a particle. On the other hand, the topological features of a particle can be inferred from the surface scattering *via* Power-law with two variables, *B*_*i*_ and *d*_*f*_, at higher *q*. *B*_*i*_ is directly proportional to the surface area of the particle while *d*_*f*_ provides a measure of mass to size scaling or density of an average particle. These local scattering laws can be combined using the Unified Function to describe multiple levels of structures. The choice of the number of the levels used for the data interpretation depends on the features in the SANS curve. Owing to the presence of three distinct bend/hump in the SANS curve (at *q* ~ 0.004, 0.02 and 0.07 Å^−1^), the GOx dimer system is fitted with a three level Unified Fit, as:^2,18,44,46–49^

1 $$\begin{array}{rcl}I(q) & = & \{{G}_{c}{e}^{(\frac{-{q}^{2}{R}_{g,c}^{2}}{3})}+{B}_{c}{e}^{(\frac{-{q}^{2}{R}_{g,a}^{2}}{3})}{({q}_{c}^{\ast })}^{-4}\}\\  &  & +\{{G}_{a}{e}^{(\frac{-{q}^{2}{R}_{g,a}^{2}}{3})}+{B}_{a}{e}^{(\frac{-{q}^{2}{R}_{g,G}^{2}}{3})}{({q}_{a}^{\ast })}^{-{d}_{f}}\}\\  &  & +\{{G}_{G}{e}^{(\frac{-{q}^{2}{R}_{g,G}^{2}}{3})}+{B}_{G}{({q}_{G}^{\ast })}^{-{d}_{G}}\}\end{array}$$

where, ${q}_{i}^{\ast }={q/\{erf(q{k}_{sc}{R}_{g,i}/\sqrt{6})\}}^{3}$, ${k}_{sc}\approx 1.06$, and *erf* is the error function. The subscripts *c*, *a* and *G* indicate different levels of GOx organizations namely the clusters, aggregates and GOx dimer levels, respectively. The power–law prefactor^18,19,43,44,50^,

2 $${B}_{i}={n}_{i}{S}_{i}\Delta {\rho }^{2}$$

where, *n*_*i*_ and *S*_*i*_ are the number and surface area of particles in a structural level while Δ*ρ* is the contrast factor. The power law exponent, more widely called the fractal dimension, is the mass to size scaling parameter. It is a direct measure of density whence a higher fractal dimension translates to a higher mass density. The Guinier prefactors^20,43,44,50^

3 $${G}_{i}={n}_{i}{{V}_{i}}^{2}\Delta {\rho }^{2}$$

where, ${V}_{i}$ is the volume of particles in a structural level. Few GOx dimers constitute a larger average aggregate and several of such aggregates then form much larger clusters. Since the contrast, Δ*ρ*, remains the same across all the levels, the relation

4 $$\frac{{G}_{a}}{{G}_{G}}=\frac{{n}_{a}{{V}_{a}}^{2}}{{n}_{G}{{V}_{G}}^{2}}=\frac{{V}_{a}}{{V}_{G}}={z}_{a}$$

gives the number of dimers in an average aggregate, or the degree of aggregation in the aggregate structure. Similarly the number of these aggregates in the cluster can be evaluated by^47^,

5 $${z}_{c}=\frac{{G}_{c}}{{G}_{a}}$$

The ${z}_{i}\text{'}s$ are independent of absolute data calibration with the assumption that the ${i}^{th}$ level is composed of ${(i-1)}^{th}$ level structures.

### *Ab initio* modeling of SANS Data

Small-angle scattering techniques such as SANS are widely used to evaluate three dimensional protein structures by implementing *ab initio* modeling approaches such as the DAMMIN program in the ATSAS suite, developed by Svergun’s group^20,21^. The program generates dummy bead coordinates of structures consistent with isotropic SANS experimental data^51^ using finite volume ensembles with the help of parameterized envelope functions. The algorithm used to generate the individual volume elements is bound by constraints of compactness, which takes size and distribution inputs from GNOM fits of SANS data^21,52^. The GNOM program can be conveniently used to fit single structural levels but presently has limitations with respect to hierarchical topologies.

In the present study, the total scattering intensity has been separated into a set of three distinct intensities such that,

6 $$I(q)={I}_{c}(q)+{I}_{a}(q)+{I}_{G}(q)$$

where,

7 $${I}_{c}(q)={G}_{c}{e}^{(\frac{-{q}^{2}{R}_{g,c}^{2}}{3})}+{B}_{c}{e}^{(\frac{-{q}^{2}{R}_{g,a}^{2}}{3})}{({q}_{c}^{\ast })}^{-4}$$

8 $${I}_{a}(q)={G}_{a}{e}^{(\frac{-{q}^{2}{R}_{g,a}^{2}}{3})}+{B}_{a}{e}^{(\frac{-{q}^{2}{R}_{g,G}^{2}}{3})}{({q}_{a}^{\ast })}^{-{d}_{f}}$$

9 $${I}_{G}(q)=I(q)-{I}_{c}(q)-{I}_{a}(q)$$

The GOx enzyme level scattering can therefore be obtained using the contribution from GOx level using ${I}_{G}(q)$ as given by Eq. (9). Error bars, ${s}_{i}(q)$ for each of the scattering intensities, ${I}_{i}(q)$, were generated by normalization with respect to the experimental SANS intensity errors,

10 $${s}_{i}(q)=\sqrt{{{s}^{2}}_{\exp }(q)\frac{{I}_{i}(q)}{{I}_{exp}(q)}}$$

where, ${s}_{exp}(q)$ is the error from the experimental SANS intensity, ${I}_{exp}(q)$.

For *ab initio* modeling, the size ranges of the larger hierarchical structures were scaled to that of the GOx dimer in order to limit the search volume to generate appropriate dummy atom models using the GNOM fits. This was achieved by scaling the *q*-range of cluster and aggregate structures as,

11 $${q^{\prime} }_{i}={q}_{i}\frac{{R}_{g,G}}{{R}_{g,i}}$$

The result of application of Eqs (10, 11) are shown in Fig. 1(c,d) for SANS data from sample 1 and samples 2 respectively. In the present study, the intensities obtained from using Eqs (7–9) were fit using GNOM which uses the distance distribution function, *p*(*r*), that may be calculated from the intensity using a Fourier transform,

12 $$p(r)=\frac{{r}^{2}}{2{\pi }^{2}}{\int }_{0}^{\infty }I(q)\frac{sin(qr)}{qr}{q}^{2}dq$$

In practice, the integration is over *r* = 0 to a maximum distance $r={D}_{max}$. In general, the *D*_max_ is selected in the range of 2.5–3 times *R*_*g*_, depending on the feasibility of shape of the distribution function. We started with a D_max_ of 70 and adjusted thereafter as required. The *P(r)* plots for the two samples for the GOx, aggregate and cluster levels are shown in the Fig. S3. The details of the *D*_*max*_ are in the Table S1. The GNOM^52^ output file is used to generate 20 low-resolution *ab initio* models using the DAMMIN program^21^. Thereafter, these set of 20 *ab initio* models were sorted, aligned and averaged using the DAMAVER program suite to generate final models^53^. Table S1 has the statistical details of the *ab-initio* modeling using ATSAS package including the normalized spatial discrepancy (NSD), the number of reconstruction included in the final model generated by DAMAVER and Dmax used for generating GNOM fits.

The ability of differentiating and effectively outlining the thermodynamics and topologies in fractal and hierarchical structure using Unified Fit on SANS is well investigated^18,43,49^.

### Integration of multi-resolution structures

To effectively characterize the structure of immobilized GOx, a three-dimensional volumetric map was generated from the averaged model using the pdb2vol module of the Situs software package^54^. Subsequently a rigid-body docking approach was employed to perform exhaustive six-dimension conformational searches (3 rotations and 3 translations) to fit the high-resolution crystallographic model (PDB ID: 1GPE^55^ at 1.8 Å resolution and a Diffraction Precision Index of 0.1 Å^56,57^) into the modeled low-resolution averaged molecular envelopes. The entire rigid-body docking procedure was automated using the CoLoRes^54^ module of Situs. This cross-correlation function-based module implements fast Fourier transform algorithms and Laplacian filter for an effective computation of the degrees of freedom of the crystallographic model corresponding to the fixed molecular envelope. The docking was performed with an angular step of 20, followed by Powell minimization.

### Evaluation of electrostatic potential

The electrostatic potentials (EPs) were evaluated using Adaptive Poison-Boltzmann Solver (APBS) and PBD2PQR software package which utilizes the continuum electrostatics calculations^58^. The high resolution crystallographic model (PDB ID: 1GPE^55^) was used with PARSE forcefield optimized for examining models under implicit solvent conditions of pH 7 and pH 9^58^. The APBS solves a linearized form of Poisson-Boltzmann equation to evaluate the EP, which have been visualized using Jmol PBD2PQR on a linear red-green-blue colour scale representing EPs of −7.5−0*K*_*B*_*T*/*e* as shown in Fig. 3(i,j).

## Supplementary information

Supporting Information: Structural determination of Enzyme-Graphene Nanocomposite Sensor Material
