# Supplementary material for: Structural determination of Enzyme-Graphene Nanocomposite Sensor Material
Source: Sci Rep. 2019 Oct 29;9:15519. doi: 10.1038/s41598-019-51882-7 (PMC6820869; doi:10.1038/s41598-019-51882-7)
Supplement: Supplementary file 1 — Supporting Information: Structural determination of Enzyme-Graphene Nanocomposite Sensor Material [file 41598_2019_51882_MOESM1_ESM.docx]

**Supporting Information: Structural determination of Enzyme-Graphene Nanocomposite Sensor Material**

Durgesh K. Rai^1^**^*^**, Manickam Gurusaran^2^, Volker Urban^3^**^#^**, Kiana Aran^4^, Lulu Ma^5^, Pingzuo Li^6^**,** Shuo Qian^3^, Tharangattu N. Narayanan^7^, Pulickel M. Ajayan^5^, Dorian Liepmann^4^, Kanagaraj Sekar^8^, María-Efigenia Álvarez-Cao^9^, Juan-José Escuder-Rodríguez^9^, María-Esperanza Cerdán^9^, María-Isabel González-Siso^9^, Sowmya Viswanathan^10^, Ramasamy Paulmurugan^11^, Venkatesan Renugopalakrishnan^6,12,^**^‡^**

*^1^Cornell High Energy Synchrotron Source, Cornell University, Ithaca, New York-14853, USA*

*^2^Institute for Cell and Molecular Biosciences, Newcastle University, Newcastle upon Tyne-NE1 7RU, UK*

*^3^Neutron Scattering Division, Oak Ridge National Laboratory, Oak Ridge, Tennessee-37831, USA*

*^4^Department of Bioengineering, University of California, Berkeley, Berkeley, California-94709, USA*

*^5^Department of Mechanical Engineering and Materials Science, Rice University, Houston, Texas-77005, USA*

*^6^Center for Life Sciences, Boston Children’s Hospital, Harvard Medical School, Boston, Massachusetts-02115, USA*

*^7^Tata Institute of Fundamental Research – Center for Interdisciplinary Sciences, Hyderabad-500107, India*

*^8^Department of Computational and Data Sciences, Indian Institute of Science, Bangalore-560012, India*

*^9^Universidade da Coruña, Grupo EXPRELA, F. Ciencias & Centro de Investigacións Científicas Avanzadas (CICA) & Instituto de Investigación Biomédica A Coruña (INIBIC). A Coruña-15011, Spain*

*^10^Newton Wellesley Hospital / Partners Healthcare System, Newton, Massachusetts-02462, USA*

*^11^Cellular Pathway Imaging Laboratory (CPIL), Dept. of Radiology, Stanford University School of Medicine, 3155 Porter Drive, Suite 2236, Palo Alto, California-94304, USA*

*^12^Department of Chemistry and Chemical Biology, Northeastern University, Boston, Massachusetts-02115, USA*

^*^Email: [dkr42@cornell.edu](mailto:dkr42@cornell.edu)

^#^Email: [urbanvs@ornl.gov](mailto:urbanvs@ornl.gov)

**^‡^**Email: [v.renugopalakrishnan@northeastern.edu](mailto:v.renugopalakrishnan@northeastern.edu)

*Inspired by Varun, Suraj*

**Supporting Figures:**


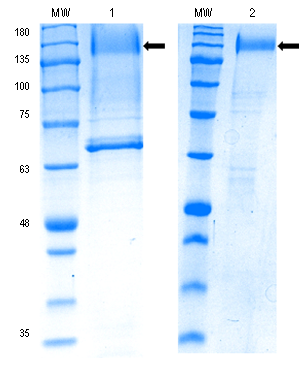


***Figure S1:*** *Coomassie blue stained SDS-PAGE analysis of the Aspergillus niger glucose oxidase. Protein is highly glycosylated and migrates as a broad band indicated by an arrow. Lane 1, Medium Grade Recombinant GOx (produced by Klyuveromyces marxianus); lane 2, High Grade Recombinant GOx (Produced by Klyuveromyces marxianus); MW, molecular-weight size marker. Each lane contains 3µg of total protein.*


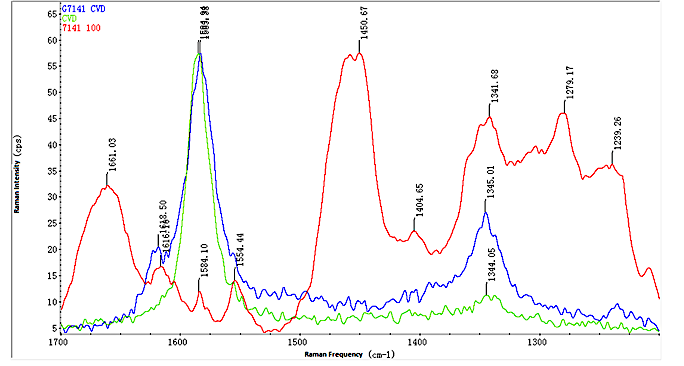


***Figure S2:*** *Raman spectrum of GOx immobilized on single layer graphene (SLG, GT141 CVD) collected at ~ pH 7 (blue), after subtraction of SLG (CVD).*


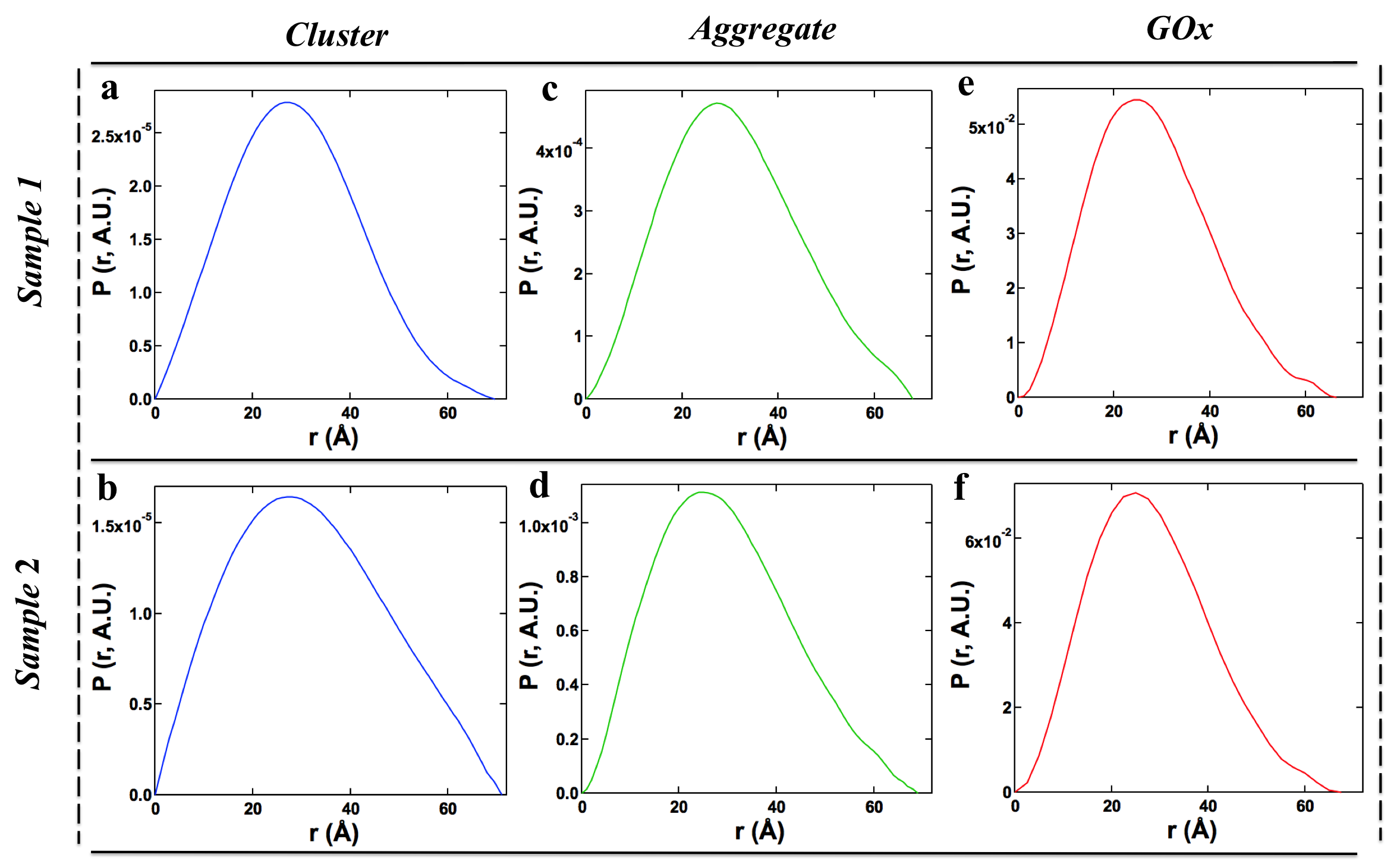


***Figure S3****: Probability distribution, P(r), plots using equation (12) for (a) sample 1 and (b) sample 2 GOx dimers; (c) sample 1 and (d) sample 2 aggregates and (e) sample 1 and (f) sample 2 clusters.*

***Table S1:*** *Statistical details of the ab-initio modeling using ATSAS package.*

| *Sample* | *Level* | *NSD**  *(Std Deviation)* | *Reconstructions included by DAMAVER* | *Dmax (GNOM, Å)* | *Dmax (DAMAVER, Å)* |
| --- | --- | --- | --- | --- | --- |
| *1* | *GOx* | *0.58*  *(±0.06)* | *18/20* | *69.6* | *69.2* |
|  | *Aggregate* | *0.69*  *(±0.06)* | *19/20* | *68* | *71.6* |
|  | *Cluster* | *0.480*  *(±0.007)* | *19/20* | *66.5* | *76.3* |
| *2* | *GOx* | *0.66*  *(±0.12)* | *18/20* | *71* | *72.7* |
|  | *Aggregate* | *0.87*  *(±0.04)* | *18/20* | *69* | *75.7* |
|  | *Cluster* | *0.488*  *(±0.007)* | *20/20* | *67.5* | *78.7* |

**Normalized spatial discrepancy (NSD)*
